# Evaluation of the DN-Mini (miniaturized double net) trap for sampling host-seeking *Anopheles* mosquitoes in malaria-endemic villages of southern Tanzania

**DOI:** 10.1371/journal.pone.0294192

**Published:** 2024-02-14

**Authors:** Alex J. Limwagu, Betwel J. Msugupakulya, Masoud M. Kilalangongono, Yohana A. Mwalugelo, Fredros O. Okumu, Issa N. Lyimo, Billy E. Ngasala

**Affiliations:** 1 Environmental Health and Ecological Science Department, Ifakara Health Institute, Morogoro, Tanzania; 2 Parasitology and Medical Entomology Department, Muhimbili University of Health and Allied Science, Dar es Salaam, Tanzania; 3 Department of Vector Biology, Liverpool School of Tropical Medicine, Liverpool, United Kingdom; 4 Department of Biomedical Sciences, Jaramogi Oginga Odinga University of Science and Technology, Bondo, Kenya; 5 School of Life Science and Bioengineering, Nelson Mandela Africa Institution of Science and Technology, Arusha, Tanzania; 6 School of Biodiversity, One Health, and Veterinary Medicine, University of Glasgow, Glasgow, United Kingdom; Clinton Health Access Initiative, UNITED STATES

## Abstract

**Background:**

Surveillance of malaria vectors is crucial for assessing the transmission risk and impact of control measures. Human landing catches (HLC) directly estimate the biting rates but raise ethical concerns due to the exposure of volunteers to mosquito-borne pathogens. A common alternative is the CDC-light trap, which is effective for catching host-seeking mosquitoes indoors but not outdoors. New, exposure-free methods are needed for sampling mosquitoes indoors and outdoors in ways that reflect their natural risk profiles. The aim of this study was therefore to evaluate the efficacy of the miniaturized double net trap (DN-Mini) for sampling host-seeking mosquitoes in south-eastern Tanzania, where malaria transmission is dominated by *Anopheles funestus*.

**Methods:**

Adult mosquitoes were collected from 222 randomly selected houses across three villages (74 per village) in Ulanga district, south-eastern Tanzania, using the DN-Mini traps, CDC-Light traps, and Prokopack aspirators. First, we compared CDC-light and DN-Mini traps for collecting indoor host-seeking mosquitoes, while Prokopack aspirators were used for indoor-resting mosquitoes. Second, we deployed the DN-Mini and Prokopack aspirators to collect host-seeking and resting mosquitoes indoors and outdoors. Generalized linear mixed models (GLMM) with a negative binomial distribution were used to compare the effectiveness of the traps for catching different mosquito species.

**Results:**

The DN-Mini was 1.53 times more efficient in collecting *An*. *funestus* indoors (RR = 1.53, 95% CI: 1.190–1.98) compared to the CDC-Light trap. However, for *Anopheles arabiensis*, the DN-Mini caught only 0.32 times as many mosquitoes indoors as the CDC-Light traps (RR = 0.32, 95% CI: 0.183–0.567). Both *An*. *funestus* and *An*. *arabiensis* were found to be more abundant indoors than outdoors when collected using the DN-Mini trap. Similarly, the Prokopack aspirator was greater indoors than outdoors for both *An*. *funestus* and *An*. *arabiensis*.

**Conclusion:**

The DN-Mini outperformed the CDC-light trap in sampling the dominant malaria vector, *An*. *funestus* species, but was less effective in capturing An. *arabiensis*, and for both vector species, the biting risk was greater indoors than outdoors when measured using the DN-Mini trap. These findings highlight the importance of selecting appropriate trapping methods based on mosquito species and behaviors.

## Introduction

Entomological indicators of malaria are key metrics used to assess the burden of malaria transmission and evaluate the effectiveness of vector control interventions. Furthermore, the development and implementation of effective interventions require in-depth knowledge of the local vector species on their vector species composition, distribution, abundance, feeding behaviors, host preference, parity status, human biting rates and pathogen infection rates [1]. To generating such knowledge requires reliable and standardized efficient mosquito sampling techniques for mosquitoes in their different physiological states, e.g., host-seeking and resting populations. Unfortunately, the standardized methods/tools for estimating these important parameters has been a major impediment [2, 3] especially in settings where substantial malaria transmission occurs outdoors [4].

Human Landing Catches (HLC), where volunteers expose their legs and catch mosquitoes landing on them, is the most direct method for measuring human biting rates and is constantly used as a reference when evaluating other sampling techniques [5]. One advantage of using HLC is that the mosquitoes are caught in the act of attacking the human host [1, 6]. The collected mosquitoes are considered representative of the natural human biting rates and are used to understand pathogen transmission risk. On the other hand, the main weaknesses of HLC include high costs, laboriousness, and the potential exposure of the volunteers to mosquito bites and mosquito-borne diseases (e.g., dengue fever, Zika, malaria, chikungunya, and others). While other studies suggest that proper use of HLC may reduce the risks of pathogen transmission to volunteers [7, 8]. Therefore, there is a strong need for alternative mosquito sampling methods that enable entomological surveillance without exposing human volunteers to mosquito bites and the risks of pathogen transmission. Various alternative methods for collecting mosquitoes have been developed [6, 9, 10], but CDC light traps have gained the most popularity due to their reliability, portability, cost-effectiveness, and scalability [11–14]. While CDC light traps are commonly used indoors alongside human-occupied bed nets and are considered more sensitive [6], their effectiveness diminishes significantly when used outdoors [15, 16].

**Fig 1:**
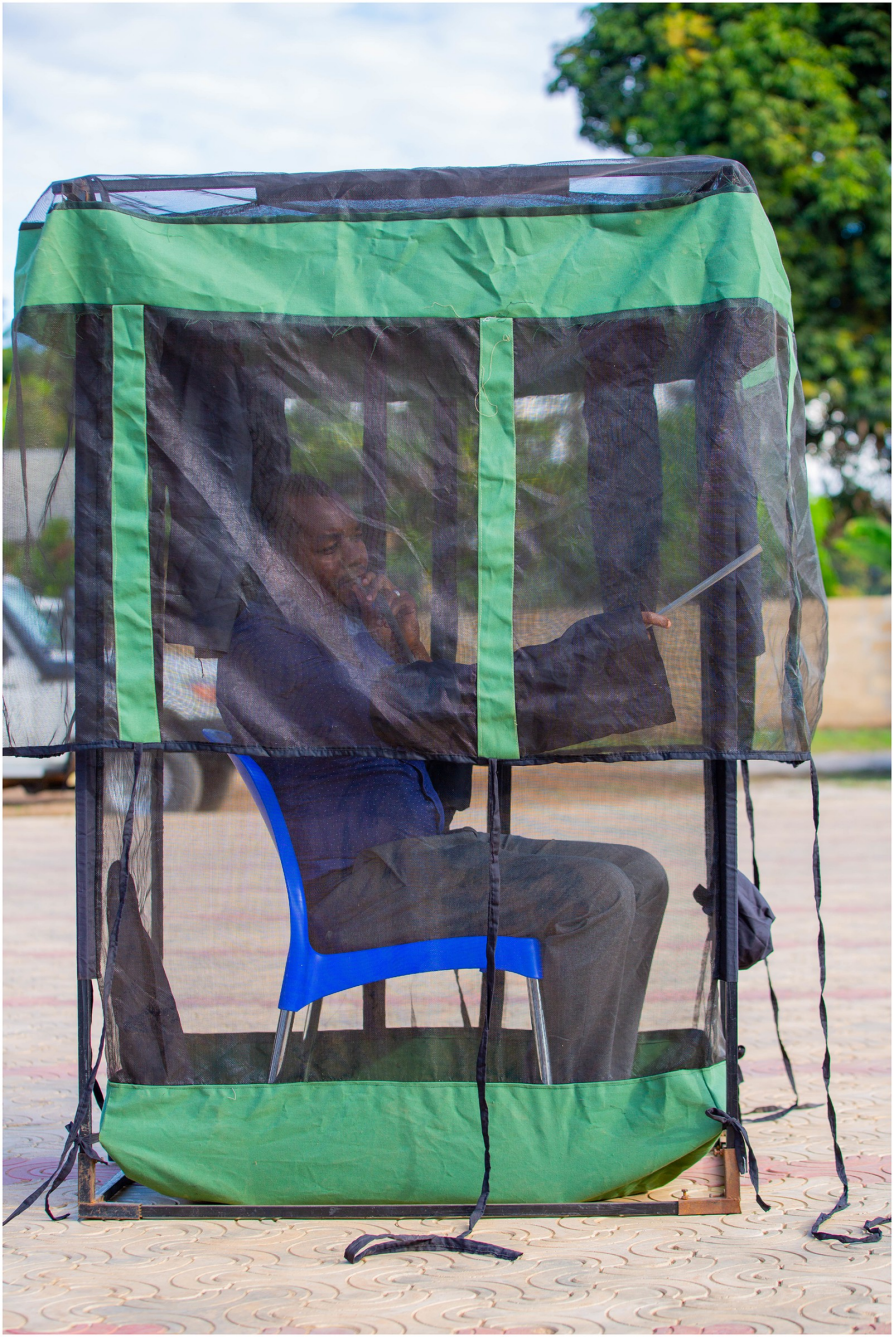
Showing a miniaturized double net trap (DN-Mini trap).

In response to the challenges posed by traditional mosquito sampling methods, the miniaturized double net trap (DN-Mini) was introduced in 2019 as an exposure-free alternative for capturing host-seeking mosquitoes both indoors and outdoors (Fig 1). This system is a variant of the traditional double net trap designs [1, 2], but allows for direct assessment of human biting risk without exposing the volunteers. The DN-Mini has been widely tested agais in various locations, including Zanzibar [17] and mainland Tanzania [3, 18, 19] as an ethical alternative to HLC. It is becoming a common alternative method when HLC is not acceptable due to ethical reasons [2, 5, 20]. However, limited data exist on its direct evaluation as a sampling tool, except for one study in rural Tanzania that examined its use for assessing indoor-outdoor biting preferences and physiological ages of malaria vectors [3].

The overall aim of this study was to evaluate the performance of DN-Mini traps as a sampling and surveillance tool for malaria vectors from indoors and outdoors of houses in southeastern Tanzania. The specific aims of this study include: 1) to compare the efficacy of the DN-Mini traps with that of the CDC-Light traps in capturing mosquitoes from inside houses (indoors); 2) to assess the efficacy of DN-Mini traps in sampling mosquitoes from both indoors and outdoors of houses; 3) to compare catches of resting mosquitoes in prokopack aspirators from indoors and outdoors of houses with DN-Mini traps and CDC-Light traps.

## Methods

### Study area

**Fig 2:**
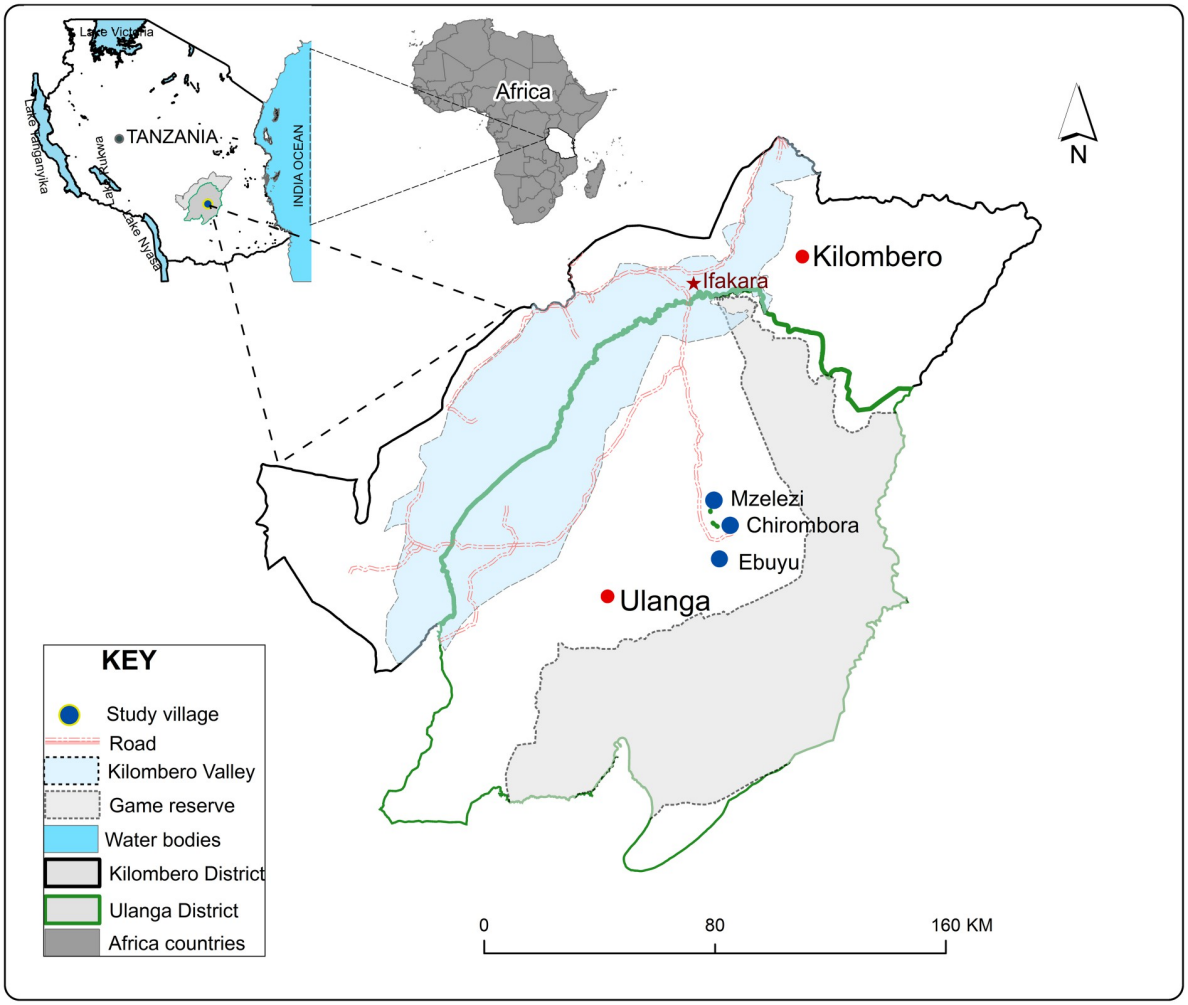
Map of the study area, showing study villages.

The study was conducted in Ulanga district, Morogoro region, southeastern Tanzania (Fig 2). The three villages involved were Mzelezi (-8.898 S, 36.735 E), Chirombola (-8.926 S, 36.753 E), and Ebuyu (-8.979 S, 36.760 E), all situated 500m above sea level. The area experiences an annual rainfall between 1200 and 1800mm, with a hot and humid season (December to May), followed by a cool-dry period (June–July), and a hot dry season (August–November). The main economic activities in the area are maize and rice farming. Furthermore, *Anopheles arabiensis* and *An*. *funestus* stands as the principal malaria vector in the study area. The prevalent housing structures typically include clay brick walls, open eaves, and open windows. Malaria transmission is moderate to high and mainly mediated by *An*. *funestus*. [11, 21].

### The miniaturized double net (DN-Mini) trap

**Fig 3:**
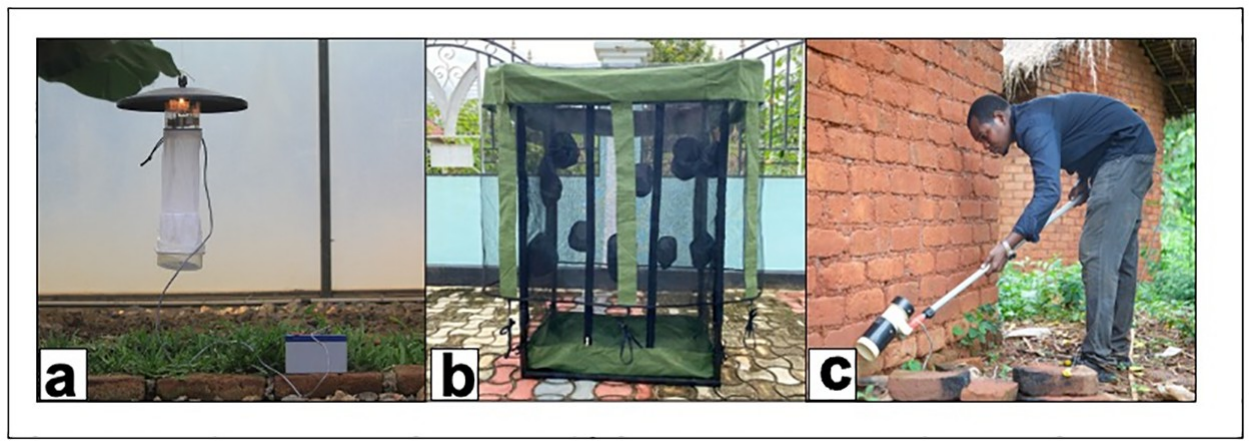
Illustrations of Mosquito Traps **A)** Standard CDC-LT (Model 512; John W. Hock Company, Gainesville, FL) **B)** A miniaturized double net trap (DN-Mini). **C)** Prokopack aspirator (John W. Hock Company model 1412).

The DN-Mini Trap is an exposure-free mosquito sampling tool designed to capture mosquitoes indoors and outdoors without interfering with their natural behavior (Fig 3). It is based on the original bed net system [1], also used in Lao PDR by Tangena *et al*. [2]. The trap comprises a fiberglass-netted cage with canvas-reinforced corners and a Polyvinyl Chloride (PVC) base, where a human volunteer sits as bait for mosquitoes. Multiple protective sleeves on the inner wall allow volunteers to safely retrieve captured mosquitoes using mouth aspirators. Mosquitoes enter the trap through the gap between the two nets and are collected at regular intervals (Fig 3). The trap’s dimensions are 60 in cm width, 100 cm in length, and 180 cm in height [3, 22]. It is made of UV-resistant netting in an iron-framed cage. The current version costs approximately 100 USD per unit per year and is locally available.

The trap can be easily assembled and dismantled, requiring less than five minutes for setup and takedown. It is low-maintenance, with minimal repairs needed in case of damage during data collection. To ensure its longevity, the trap should be stored away from direct sunlight to protect the netting fibers. Handling should pay particular attention when entering or exiting the trap, as damage typically occurs when the chair inside the trap rubs tightly on the walls, affecting the edges of the nets fitted to iron poles.

### CDC-light trap

The Centers for Disease Control and Prevention (CDC) light traps (John W. Hock Ltd, Gainesville, FL., USA) (Fig 3) are portable devices comprising a light source, a fan, and a collection container. Mosquitoes are attracted to the light and drawn into the trap by the fan. While commonly used for collecting indoor mosquitoes [16, 23, 24], they can also be applied outdoors alone [5] or when combined with other traps. It is one of the most common sampling tools for *Anopheles* mosquitoes in Africa today.

### Prokopack aspirator

The Prokopack aspirator (John W. Hock Company model 1412) [25] is a portable and user-friendly trap designed for sampling resting mosquitoes indoors and outdoors. It has a cylindrical shape housing a fan powered by 12-volt batteries. The fan pulls and sucks mosquitoes into the wire mesh surface, where they are collected until the fan is turned off and then packed into a collection bag or paper cup. This trap is designed for collecting both indoor and outdoor resting mosquitoes (Fig 3).

### Study design

This cross-sectional entomological survey was conducted in three purposefully selected villages: Mzelezi, Chirombora, and Ebuyu, in Ulanga district, southeastern Tanzania (Fig 2). These villages are approximately 70km from Ifakara town, where the Ifakara Health Institute offices are located. We sampled mosquitoes in a total of 222 households that were systematically randomly selected in three villages, each with 74 houses (74 houses/villages x 3 villages = 222 houses). Among these 74 households per village, 36 houses were randomly assigned to DN-Mini traps for random mosquito collections as random households, and 1 house was assigned as a sentinel household; the other 36 houses were assigned to CDC-Traps for random mosquito collections (random households), and 1 house was assigned as a sentinel household. The mosquito catches from random households enable us to control for the spatial variations to compare the efficacy of CDC traps and DN-Mini traps in collecting host-seeking malaria vectors from inside houses (indoors), and those from sentinel households monitor the variations in catches and vector behavior with time and space (temporal and spatial variations) of mosquitoes collected through CDC traps, DN-Mini traps, and Prokopack aspirators from inside (indoors) and outside (outdoors) houses. Each of these households was 30 meters from one another to avoid competition between traps. Mosquito collections were conducted for three consecutive days, with a one-day interval between each collection day, resulting in three days of collections within a single week. A total of three households were covered each day of mosquito collection, including one sentinel and two random households. Each time of collection of host-seeking mosquitoes from random households, mosquitoes were collected from two houses per day and moved to the other two different houses until 36 houses were covered for DN-Mini traps and those for CDC-Traps (2 houses per day x 18 days = 36 houses for each trap design). Additionally, the collection of host-seeking mosquitoes from sentinel households using the DN-Mini Trap from inside (indoors) and outside (outdoor) houses was performed repeatedly from the same house once per week for 16 weeks (1 day per week x 16 weeks = 16 days/nights). The resting mosquitoes were also collected from sentinel households using Prokopack aspirators once per week for 16 weeks from inside and outside the house (1 day per week x 16 weeks = 16 days). The GPS coordinates (latitudes and longitudes) of random and sentinel households were collected using a Garmin eTrex 20GPS device.

### Mosquito collection

During the high malaria transmission period (March to July) in 2022, adult mosquitoes were collected monthly in each village by eight trained volunteers (Fig 2).

### Comparison of the DN-Mini trap against CDC light trap

Host-seeking mosquitoes were collected indoors for three days each week for different 74 houses, 37 households for the DN-Mini trap and CDC light traps (1 sentinel and 36 random households). The CDC light trap was placed in the sleeping room about 1.5 meters from the floor surface, close to the feet of household member sleeping under treated bed net [26]. The DN-Mini trap was positioned in the sitting room, and was occupied by an adult volunteer, who also retrieved mosquitoes periodically as previously described [3]. Both traps CDC-Light trap and DN-Mini trap were set at 18:00 hrs and mosquitoes retrieved at 6:00 hrs the following morning.

### Mosquito composition between indoors and outdoors biting

Two pairs of human volunteers in a DN-Mini trap were used for the collection of indoor and outdoor mosquito compositions. One DN-Mini trap was placed indoors in the sitting room and another outdoors, five meters away from the house. In these surveys, the DN-Mini traps were used to collect mosquitoes every one-hour interval between 18:00 hrs. and 6:00 hrs. the following morning. Mosquitoes collection was done once a week for four months at the sentinel houses (1 sentinel house/village x 3 villages = 3 houses/day).

### Composition of resting mosquitoes

Resting mosquitoes were collected indoors from ceilings and walls and outdoors around five meters from the sampled house from flowerpots, plants, walls, and general outdoor surfaces using the Prokopack aspirator. Resting mosquitoes were collected indoors and outdoors to assess the composition of mosquitoes and whether the species composition captured by host-seeking mosquitoes matches the composition resting indoors. The same person who collected mosquitoes from the DN-Mini traps was assigned to perform the Prokopack aspirators once a week, between 6:00 a.m. and 7:30 a.m., for the duration of four months at the sentinel houses to which the DN-Mini trap had been assigned.

### Mosquito identification and parasite detection

All the collected mosquitoes were killed each morning using petroleum or alcohol fumes, then sorted by taxa and sex using the keys for morphological mosquito identification [27]. The abdominal status of each female *Anopheles* mosquitoes was recorded as blood fed, unfed, partially fed or semi-gravid or gravid. Female *Anopheles* mosquitoes were packaged individually or in pools in microcentrifuge (Eppendorf^®^) tubes filled with silica gel as a preservative. Each tube was provided with a unique identification number and packed in the storage boxes labeled with village name, house number, trap position, mosquito species name and date of collection; then submitted for further laboratory analysis. Additionally, a sub-sample of female malaria vectors, *An*. *gambiae s*.*l* were examined by multiplex polymerase chain reaction (PCR) for species identification to confirm and distinguish the sibling species [28]. A sub sample of *An*. *funestus s*.*l*. were also examined by PCR, using a technique adapted from Koekemoer *at al* [29] and Cohuet *at al* [30], to identify sibling species in *An*. *funestus* group. Furthermore, the head and thorax of *Anopheles* mosquitoes were separated from abdomen and tested for the presence of *Plasmodium falciparum* circum-sporozoite protein (*Pf* CSP) using the enzyme-linked immunosorbent assay (ELISA) method [29].

### Data analysis

Data were analyzed using R open-source statistical software version 4.2.1 [31]. Descriptive statistics were used to summarize the data using frequencies, means and proportions. Generalized linear mixed models (GLMM) following negative binomial distribution was implemented using *lme4* package [31], to assess the relationship between number of mosquitoes collected and different trap types. The number of mosquitoes collected were modeled as a response variable while mosquito traps and villages were added as fixed factors. To take into account of the variations in the mosquito catches, the different days, household ID and collection date were included as random factors. Each mosquito species was analyzed separately, in this model, our focus was solely on *An*. *arabiensis*, *An*. *funestus* and *Culex* mosquitoes. These particular species were selected due to their substantial collection numbers, while vector species with fewer numbers or catches were excluded from the analysis. The relative rates of mosquito catches were obtained with their associated 95% confidence intervals and considered significant when p-values were less than or equal to the 5% significance level. The models were also used to generate the estimated marginal means (EMM) for catches in each trap using *ggeffects* package [32]. Graphs were plotted using *ggplot2* [33] package.

### Ethical clearance and informed consent

The research was conducted following the principles of the Declaration of Helsinki. Ethical approval was obtained from the Muhimbili University of Health and Allied Sciences Review Board (MUHAS-REC-12-2021-910), and permission to publish the work was granted by NIMR (Ref: NIMR/HQ/P.12VOL.XXXVI/27). Approval to conduct the study was obtained from the district medical officer of Ulanga district and the local government leadership in the selected villages. Before commencing the study, meetings were held with local government leaders to explain the study’s aim and procedures. Verbal and written informed consent was obtained from individual house occupants and human volunteers involved in mosquito collection. Participants were fully informed of potential benefits and risks, and their voluntary participation was assured. Participants were also informed of their right to withdraw from the study at any time without consequences. Confidentiality was maintained to ensure the anonymity of participants.

## Results

### Mosquito species composition and abundance

Overall, a total of 19,841 female mosquitoes were collected indoors and outdoors between March and July 2022 using CDC-light traps, DN-Mini traps, and Prokopack aspirators. About equal numbers of mosquitoes were collected between CDC light traps (45.4%, n = 9,008) and DN-Mini traps (44.0%, n = 8732), and 10.6% (n = 2101) were collected through prokopack aspirators. The majority of mosquitoes collected through the DN-Mini trap were collected indoors (89.9%, n = 7851), and 10.1% (n = 881) were collected outdoors (Table 1). *An*. *funestus* accounted for 6.1% (n = 1219), *An*. *gambiae* ss accounted for 1.6% (n = 313), and other *anopheles* species accounted for 0.1% (n = 10) (Table 1). A majority of the mosquitoes collected were of the culicine group, of which *Culex* species accounted for 92.1% (n = 18268), *Mansonia* species accounted for 0.1% (n = 29), and *Aedes* species accounted for 0.0% (n = 2) of all mosquitoes.

**Table 1:** Total number of mosquitoes species collected by the different traps.

Trap types	Vector species	Number collected (%)
CDC-Light trap indoors	*An*. *funestus s*.*l*.	399 (4.4)
	*An*. *gambiae s*.*l*.	202 (2.2)
	*An*. *coustani spp*.	4 (0.0)
	*Culex spp*.	8396 (93.2)
	*Mansonia spp*.	7 (0.1)
	*Aedes spp*.	0 (0.0)
DN-Mini trap indoors	*An*. *funestus s*.*l*.	704 (9.0)
	*An*. *gambiae s*.*l*.	67 (0.9)
	*An*.*coustani spp*.	6 (0.1)
	*Culex spp*.	7055 (89.8)
	*Mansonia spp*.	17 (0.2)
	*Aedes spp*.	2 (0.0)
DN-Mini trap outdoors	*An*. *funestus s*.*l*.	56 (6.4)
	*An*. *gambiae s*.*l*.	9 (1.0)
	*An*.*coustani spp*.	0 (0.0)
	*Culex spp*.	812 (92.1)
	*Mansonia spp*.	4 (0.5)
	*Aedes spp*.	0 (0.0)
Prokopack aspirator indoor	*An*. *funestus s*.*l*.	54 (3.1)
	*An*. *gambiae s*.*l*.	26 (1.5)
	*An*.*coustani spp*.	0 (0.0)
	*Culex spp*.	1656 (95.3)
	*Mansonia spp*.	1 (0.1)
	*Aedes spp*.	0 (0.0)
Prokopack aspirator outdoor	*An*. *funestus s*.*l*.	6 (1.6)
	*An*. *gambiae s*.*l*.	9 (2.5)
	*An*.*coustani spp*.	0 (0.0)
	*Culex spp*.	349 (95.9)
	*Mansonia spp*.	0 (0.0)
	*Aedes spp*.	0 (0.0)

### Mosquito species composition

A total of 487 female malaria vectors were submitted to the molecular laboratory for species identification, of which 419 were *An*. *funestus* s.l. and 68 were *An*. *gambiae* s.l. The overall amplification rate for the *An*. *funestus* group was 87.8% (368/419), of which 99.7% (367/368) were confirmed as *An*. *funestus* and 0.3% (1/368) were confirmed as *Anopheles rivulorum*. On the other hand, the overall amplification rate for the sub-sample of *An*. *gambiae* complex was 94.1% (64/68), of which 93.7% (60/64) were confirmed as *Anopheles arabiensis* and 6.6% (4/64) were confirmed as *Anopheles gambiae s*.*s*.

### Detection of sporozoite rate infection in malaria vectors

Out of 487 *Anopheles* mosquitoes submitted to the molecular laboratory for ELISA, 14 *Anopheles* were confirmed to be infected with *Plasmodium falciparum* sporozoites. Of these infected mosquitoes, 92.8% (n = 13) were *An*. *funestus s*.*s*., and only one mosquito, 7.2% (n = 1), was *An*. *arabiensis*.

### Performance of CDC- light trap and DN-Mini trap for indoor mosquito collection

Comparing the performance of the two traps indoors, the DN-Mini trap outperformed the CDC light trap in capturing *An*. *funestus* (RR: 1.536, p = 0.001 Table 2). On the other hand, CDC light traps outperformed DN-Mini traps in capturing *An*. *gambiae* (RR: 0.305, p<0.001 Table 2) and *Culex* species (RR: 0.710, p<0.001) (Table 2).

**Table 2:** Comparison of performance of the indoor host-seeking traps.

Species	Traps type	Number collected (%)	RR (95% CI)	P-value
*An*. *arabiensis*	CDC-Light	202 (75.1)	1	
	DN-Mini	67 (24.9)	0.305 (0.178–0.522)	<0.001
*An*. *funestus*	CDC-Light	399 (36.2)	1	
	DN-Mini	704 (63.8)	1.536 (1.204–1.958)	0.001
*Culex species*	CDC-Light	8396 (54.3)	1	
	DN-Mini	7055 (45.7)	0.710 (0.610–0.828)	<0.001

### Composition of indoor and outdoor mosquitoes by using DN-Mini trap

Overall total of mosquito composition: 78.5% (n = 204) were *An*. *funestus* collected indoors while 21.5% (n = 56) were collected outdoors; 66.6% (n = 18) were *An*. *gambiae* collected indoors while 33.3% (n = 9) were collected outdoor; other anopheline 100% (n = 2) were collected indoors; for the non-malaria vector, about 74.1% (n = 2320) were *Culex* collected indoors while 25.9% (n = 812) were collected outdoors; *Mansonia* were 73.3% (n = 11) were collected indoors, while 26.7% (n = 4) were collected outdoor; and 100% (n = 2) ware *Aedes* mosquitoes.

### Resting mosquito composition by using prokopack aspirator

Generally, there was no significant difference between *An*. *funestus* mosquitoes collected indoors and outdoors (RR: 0.351, p = 0.210), but more outdoor collections were made for *An*. *arabiensis* (RR: 1.093, p = 0.936) and *Culex* species (RR: 0.665, p = 0.194) (Table 3). Comparing resting mosquitoes collected in houses with CDC-light traps and those with DN-Mini traps, for indoor collections, there was no significant difference between all major mosquitoes collected indoors by prokopack aspirators in the house with CDC-light trap and DN-Mini rap (RR: 1.434, p = 0.597), *An*. *funestus*, *An*. *gambiae* (RR: 1.434, p = 0.545), and *Culex* species (RR: 0.677, p = 0.143) (Table 4).

**Table 3:** Resting composition of indoor and outdoor mosquitoes by using prokopack aspirators (PP).

Species	Traps position	RR	(95% CI)	P-value
*An*. *gambiae*	Indoor	1		
	Outdoor	1.093	0.124–9.624	0.936
*An*. *funestus*	Indoor	1		
	Outdoor	0.351	0.068–1.806	0.210
*Culex species*	Indoor	1		
	Outdoor	0.665	0.359–1.231	0.194

**Table 4:** Resting composition of indoor mosquitoes collected by prokopack aspirator (PP) in the house of CDC-Light trap and DN-Mini trap.

Vector Species	Trap types	RR	(95% CI)	P-value
*An*. *gambiae*	PP-CDC-Light	1		
	PP-DN-Mini	0.565	0.089–3.599	0.545
*An*. *funestus*	PP-CDC-Light	1		
	PP-DN-Mini	1.434	0.377–5.448	0.597
*Culex species*	PP-CDC-Light	1		
	PP-DN-Mini	0.677	0.401–1.142	0.143

### Comparison of feeding status between mosquitoes collected by CDC light trap and DNM trap

**Fig 4:**
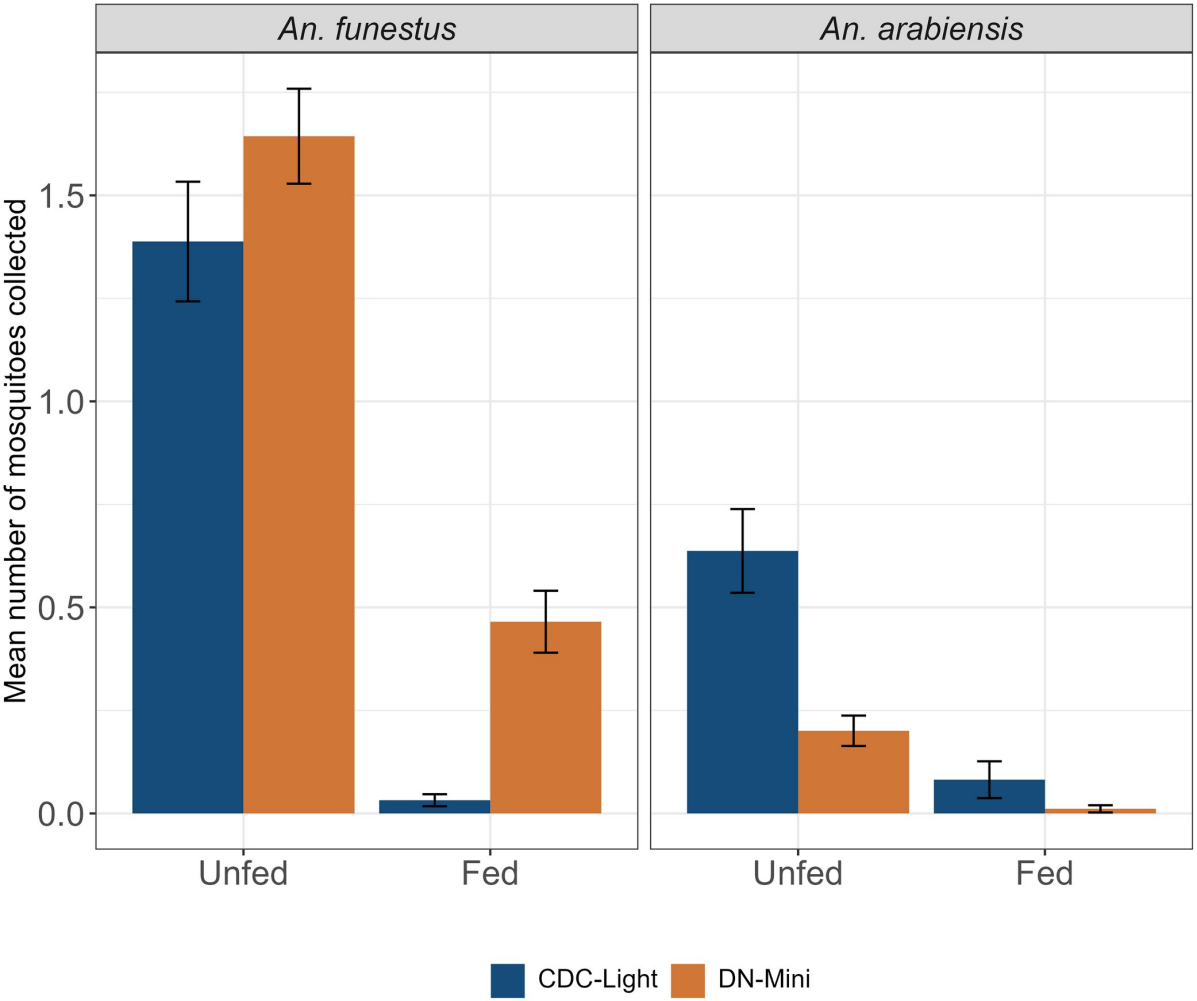
Feeding status of dominant malaria vectors caught by the CDC-light trap and the DN-Mini trap.

A total of 157 *An*. *funestus* were identified as blood-fed. Of these, 5.7% (n = 9) were collected through the CDC light traps, and 94.3% (n = 148) were collected through the DN-Mini trap. Of the 27 blood-fed *An*. *gambiae sl*, 85.2% (n = 23) were collected by a CDC-light trap, and 14.8% (n = 4) were collected by a DN-Mini trap (Fig 4).

## Discussion

In our study, the two candidate traps for the collection of host-seeking African malaria vectors, including the DN-Mini and CDC-light traps, were compared in indoor settings. And the DN Mini was also used to compare indoor and outdoor catches. The results show that the DN-Mini trap collects about twice as many *An*. *funestus* compared to the CDC-light trap indoors. This indicates that the DN-mini trap, which is baited by a sitting volunteer, can be especially efficient for sampling *An*. *funestus* mosquitoes, though it also catches other vector species. In this area, where *An*. *funestus* now dominates malaria transmission, it appears that the DN-Mini trap can be especially useful for sampling malaria vectors. On the other hand, the CDC light traps, which depend mostly on odor from people sleeping under the bed net to attract host-seeking mosquitoes, were superior in capturing *An*. *arabiensis* mosquitoes, which also contribute to malaria transmission in the region and can be especially important for outdoor transmission [14, 34]. The CDC-light trap has previously been reported to attract nearly as many *Anopheles* mosquitoes as the HLC and, in some cases, even equivalent numbers [12, 13]. The only limitations of this trap is that while it is effective for indoor collections, it is not always sufficient for outdoor collections [2]. Other studies suggest the use of CDC-light outdoor to be embedded into the other trap as an additional trap (Limwagu *et al*., unpublished).

The current study found that *An*. *funestus* is not only the most efficient malaria vector than *An*. *arabiensis*, but it is also now becoming the most abundant vector species of Anopheline mosquitoes in this study area. For example, the number of host-seeking *An*. *funestus* s.s. was 2–10 times higher than that of host-seeking *An*. *arabiensis* in indoors, but it was 6 times higher than that of host seeking *An*. *arabiensis* outdoors (Tables 1 and 2). These findings are consistent with previous studies in the Kilombero valley, which have reported *Anopheles funestus* to be responsible for over 85% of malaria transmission [35]. Our analysis of *Plasmodium* sporozoite infections also suggests that nearly all the infected mosquitoes were *An*. *funestus* and only one was *An*. *arabiensis*, thus suggesting that *An*. *funestus* dominates in both densities and malaria infection rates.

Furthermore, *An*. *funestus* could be more efficient at transmitting malaria and may be more resistant to primary control measures such as long-lasting insecticide-treated nets (LLINs) and indoor residual spray (IRS) in the study area. These interventions primarily target indoor mosquitoes, but *An*. *funestus*, which normally bite and rest indoors, are not responsive to them. Additionally, the study also found that there were higher numbers of *An*. *funestus* and *An*. *arabiensis* mosquitoes indoors compared to outdoors (Table 2). This is inconsistent with the findings of a study by Limwagu et al. [3] in the evaluation of DN-Mini traps, where *An*. *funestus* were dominant indoors while *An*. *arabiensis* were dominant outdoors. Moreover, using Prokopack aspirators, the density of *An*. *funestus* was higher indoors compared to outdoors, while for *An*. *arabiensis*, *the* majority was found outdoors compared to indoors.

The study also found that the DN-Mini trap is effective for catching mosquitoes that have recently had a blood meal, as it caught more fed mosquitoes than the CDC-light trap (Fig 4). This indicates that the mosquitoes caught by the DN-Mini trap are likely coming from a different host that had obtained a partially blood meal and are being caught by the trap while attempting to obtain a second blood meal. This could signify that the DN-Mini trap can sample host-seeking mosquitoes in the process of seeking a first or second blood meal. Hence, the HLC is a usual tool for understanding the transmission dynamic as well as host preference of mosquitoes, and such details cannot be observed from the HLC since mosquitoes collected might also feed on the HLC collector. Multiple blood feeding (taking more than one blood meal per gonotrophic cycle) was found to be the most important information when calculating human biting rate [36], such data that can easily be obtained from the DN-Mini trap. Though unlikely, the blood-fed mosquitoes may have been from the trap handlers or volunteers inside the traps. This might suggest that while the DN-Mini is designed to be exposure-free, the volunteers sitting inside the trap and retrieving the mosquitoes periodically still need to be well trained to avoid mosquito bites.

Overall, the findings taken together suggest that the DN-Mini trap can be effective for sampling malaria vectors inside houses and might, in certain cases, be even more efficient than the CDC light traps. The study also suggests that the DN Mini can be efficient for comparing indoor-outdoor biting patterns for the malaria vector species. This study therefore proves that it can be widely used for surveillance of malaria vectors across the country.

In regard to the composition of mosquitoes collected indoors and outdoors by DN-Mini trap, it was revealed that, among the anopheline mosquitoes, both *An*. *funestus* and *An*. *arabiensis* were found to be dominant, while among the culicine group, *Culex* mosquitoes were the most abundant. These results, however, appear to be inconsistent with the findings of a previous study conducted by Limwagu et al. 2019 [3]. In their study, they reported a different distribution pattern of *An*. *funestus* and *An*. *arabiensis* between indoor and outdoor collections. Limwagu et al. found that *An*. *funestus* was more prevalent indoors than outdoors. Additionally, our study revealed that *Culex* species were more dominant indoors compared to outdoors.

In resting mosquito composition by Prokopack aspirator, we found that the majority of anopheline mosquitoes were *An*. *funestus* and *An*. *arabiensis*, while *Culex* species were more abundant in the culicine group. In regards to indoor and outdoor locations, there were more *An*. *funestus* indoors, while *An*. *arabiensis* were more outdoors. Contrary to the results reported by Kreppel et al. 2020 [35], our study revealed that *An*. *funestus* found resting outdoors, while *An*. *arabiensis* exhibited a preference for outdoor resting sites.

The study conducted by Mmbando et al. 2020 [36] suggests that when employing the resting bucket (RBu), similar results are likely to be obtained, with the majority of outdoor mosquitoes being culicine and a proportion belonging to anopheline mosquitoes. It’s worth noting that the resting bucket is predominantly favored for outdoor use.

This study has certain limitations. First, the performance of the DN-Mini trap is influenced by environmental factors such as wind speed and rainfall. These factors might influence mosquito behavior and compromise trap performance, particularly when the traps are deployed outdoors. Additionally, there is a limitation related to community acceptance. When using the DN-Mini trap for indoor collection, we normally position it in living rooms. However, in some households, the spaces also serve as storage areas for harvested food items, such as bags of rice.

## Conclusion

In conclusion, the study demonstrates that the DN-Mini trap exhibits higher efficacy, being 53% more efficient in collecting *An*. *funestus* species indoors compared to the CDC-Light trap. However, for *An*. *arabiensis*, the DN-Mini trap’s efficiency is reduced, being 68% less effective indoors than the CDC-Light trap. The results also indicate that both *An*. *funestus* and *An*. *arabiensis* species are more abundant indoors than outdoors when collected using the DN-Mini trap. These findings highlight the importance of selecting appropriate trapping methods based on mosquito species and their behavioral patterns. The findings also emphasize the potential of the DN-Mini trap for malaria vector surveillance and compare indoor and outdoor biting profiles of different vector species. The DN-Mini can be particularly useful in areas such as the southeastern Tanzanian villages where *An*. *funestus* dominates transmission.
